# Water Microbiota in Greenhouses With Soilless Cultures of Tomato by Metabarcoding and Culture-Dependent Approaches

**DOI:** 10.3389/fmicb.2020.01354

**Published:** 2020-06-23

**Authors:** Adeline Picot, José F. Cobo-Díaz, Audrey Pawtowski, Christelle Donot, Fabienne Legrand, Gaétan Le Floch, Franck Déniel

**Affiliations:** Univ. Brest, Laboratoire Universitaire de Biodiversité et Écologie Microbienne, Plouzané, France

**Keywords:** water, greenhouses, microbiota, metabarcoding, biofiltration

## Abstract

Water supply, in hydroponic greenhouses, can originate from groundwater, surface water or rainwater stored in open tanks. To limit contamination of water supply, several methods have been used including active and passive methods such as slow filtration techniques which consist in passing the nutrient solutions slowly through filters. The purpose of this study was to describe the microbiota associated with water sampled before entering greenhouses and in recirculating nutrient solutions, either before or after running through a biofiltration system. Metabarcoding analysis revealed that water ecosystems were unique niches for diverse bacterial and fungal communities. Microbial composition varied greatly across storage conditions (groundwater vs. rainwater) and among greenhouses, suggesting that water microbiota is site- and storage-condition-specific. Nonetheless, we found that microbiota structure in open-stored water (either coming from ground or rain) shared a higher degree of similarity than with water directly pumped out of the ground. Open-stored waters were characterized by predominant taxa, notably those involved in aerobic chemoheterotrophy, such as the *Sphingomonadaceae* and *Hyphomicrobiaceae* families. Water directly collected from the ground showed the lowest levels of fungal and bacterial richness while also characterized by a significantly higher level of bacterial equitability and an enrichment in taxa involved in N-cycling. Slow filtration allowed reducing cultivable bacterial loads as well *Pythium* spp. and *Fusarium oxysporum* propagules, based on culture-dependent results, without compromising microbiota richness and diversity. Although compositional structure was similar following biofiltration, significant differences in bacterial (but not fungal) taxa abundance were reported, with primarily an enrichment of *Chelativorans*, *Mycobacterium*, and *Gemmata* as well as a depletion of *Rhodobacter*, *Aminobacter*, and *Ellin329*. The exact mechanisms by which such taxa would be favored at the expense of other remained unknown. Besides the accurate description of microbiota found in water at both taxonomical and predicted functional levels, our study allowed comparing the water microbiota between various storage system and following biofiltration. Although preliminary, our results provide a first insight into the potential microbial diversity, which can increase ecosystem functionality.

## Introduction

Water supply can originate from various sources in hydroponic greenhouses. It can be directly pumped out from the ground through wells or originated from surface water such as lakes and rivers. Alternatively, water tanks in which rainwater runoff are collected and stored are also commonly used as water supply. In addition, drain water can be partly recycled in soilless culture not only to comply with legislation but also to reduce environmental impacts and nutrient input although it may also cause unbalanced nutrient solutions due to ion accumulation and increase the risk of disease attacks (Lechevallier et al., 2018). In either case, water remains one of the main sources responsible for the introduction of pathogenic microorganisms, along with the substrate, the plant itself and the greenhouse environment (Rey et al., 2005). Water supply should therefore be considered as one of the hazards that may compromise hygiene and plant safety resulting in either plant infection with human pathogens such as *Salmonella* spp. or shiga toxin-producing *Escherichia coli* (Delbeke et al., 2015) or with phytopathogens (Rey et al., 2005). Notably, contamination with zoospore-producing fungi belonging to the *Pythium* and *Phytophthora* genera as well as *Fusarium oxysporum* are responsible for important water-borne diseases in soilless cultures and can be disseminated through water (Vallance et al., 2011). Surface waters, including rivers and lakes, are a potential source of fecal contamination and are frequently contaminated with microorganisms, such as enteric human pathogens, *Pythium* spp. or *F. oxysporum* (Geldreich, 1990; Stanghellini and Rasmussen, 1994; Sánchez and Gallego, 2002). Although groundwater is *a priori* of better quality than surface water, it has been evidenced that such waters, especially those derived from wells, are also subjected to microbiological contamination, including prokaryotes and eukaryotes (Geldreich, 1990; Awe et al., 2012; Gambero et al., 2017; Korbel et al., 2017).

To limit contamination of water supply in greenhouses, several methods have been used such as heat treatment, ultra-violet (UV) radiation and chemical treatments including chlorination and ozonation, resulting in an effective reduction of microbial loads (Runia, 1995; Ehret et al., 2001). These control methods have been proven efficient in many applications such as the chlorination of drinking water, introduced to the water supply in the beginning of the nineteenth-century, which undoubtedly contributed to stop the spread of pathogens, such as typhoid fever (Schoenen, 2002). However, they have been questioned when used for food production in greenhouses because these methods are non-specific and non-target microbiota, which can have protective effects against the colonization of pathogenic microorganisms, is also reduced along disinfection (Postma et al., 2000; Zhang and Tu, 2000). As a result, passive methods, such as slow filtration techniques which consist in passing the nutrient solutions slowly through filter unit filled with sand, rockwool flocks or pozzolan grains, are now preferred (Renault et al., 2018). Such physical filtration, complemented by the further colonization of filters with a complex microbiota, accounted for the successful control of various plant pathogens including bacteria, fungi, nematodes and viruses (Rey et al., 1999; Van et al., 1999; Ehret et al., 2001). However, which members of the microbiota exert suppressive effects over pathogenic microorganisms and how is not fully understood. In order to enhance the efficacy of pathogenic microorganisms removal, filters were, prior to use, amended with bacterial strains, selected for their antagonistic activity and/or plant-growth promoting activities (e.g., *Pseudomonas putida* or *Bacillus cereus*) (Brand and Wohanka, 2001; Renault et al., 2007).

Microbial diversity in water has been initially described using culture-dependent techniques (Berkelmann et al., 1994) before the development of culture-independent methods, including fingerprinting methods such as DGGE (Denaturing-Gradient Gel Electrophoresis) or SSCP (Single Strand Conformation Polymorphism) (Calvo-Bado et al., 2006; Renault et al., 2012). More recently, the use of next-generation sequencing (NGS) technologies, including metabarcoding, allows to gain a more complete picture of complex microbiota and more rapidly than fingerprinting methods. Besides the description of taxa within an ecosystem, metabarcoding techniques have also been recently used for predicting the functionality of community members (Langille et al., 2013; Tedersoo et al., 2015; Bouchez et al., 2016; Louca et al., 2016). To date, investigation of the fungal and bacterial communities associated with water in greenhouses is very limited, mostly dedicated to nutrient recirculating solutions and culture substrates (Meot-Duros et al., 2011) and has rarely been achieved through NGS approaches, with the exception of the study of López-Leal et al. (2018). These authors sequenced a water drain metagenome from one sample collected from an experimental greenhouse using a shotgun sequencing strategy. In addition, no study has been undertaken to compare microbiota between rainwater and well waters, both of which are commonly used in greenhouses as water supply.

The purpose of this study was to describe the fungal and bacterial diversity of water collected in 3 commercial hydroponic tomato greenhouses. To address this objective, culture-dependent and MiSeq Illumina metabarcoding approaches were used. Besides the accurate description of bacteria and fungi found in water at both taxonomical and predicted functional levels, our goal was also to gain a preliminary insight into taxa distribution between water samples collected from various storage systems (rainwater and groundwater either directly pumped out of the ground or stored in open tank) and before and after running through a biofiltration system.

## Materials and Methods

### Water Sampling

Water samples were collected from 3 commercial hydroponic tomato greenhouses in North Finistère in Brittany, France the 3rd or 4th of April 2017. Greenhouse 1 and 2 are 52 km apart vs. 62 km apart for greenhouse 1 and 3 and 14 km apart for greenhouse 2 and 3.

The origin of water samples and their storage conditions are reported in Table 1. In greenhouses 1 and 2, 500 mL of water were collected in sterile containers. In greenhouse 3, water was sampled before (500 mL) and after (700 mL) running through a biofiltration system, put in place since March 2015. Biofilters were amended with a mixture of 2 strains of *Pseudomonas putida* and one strain of *P. fluorescens* provided by Biovitis SA (Déniel et al., 2004, 2010). The biofiltration system (Squiban, France) held a capacity of 26 tons of media made out of pozzolan grains (about 4 mm in diameter) with a flow rate of 6,000 L/h. The nutrient solution for tomato roots running through the biofiltration system was stored in a big tank outdoor. Water of this nutrient solution originated from groundwater.

**TABLE 1:** Information about water samples collected at the beginning of April 2017 in tomato greenhouses located in the North Finistère, Brittany, France.

**Code**	**Origin of water**	**Storage conditions**	**GPS latitude-longitude coordinates of greenhouses**
GW/S	Stored groundwater	Groundwater being pumped out and stored in an open tank	Greenhouse 1 48.1070335 N -4.0225663 W
GW/NS	Non-stored groundwater	Non-stored water directly pumped out from the ground	Greenhouse 2 48.3744181 N -4.3961437 W
RW	Rainwater	Stored in an open tank	
BBF	Before biofiltration	Before running through a biofiltration system located at the entrance of the greenhouse	Greenhouse 3 48.4552172 N -4.4372351 W
ABF	After Biofiltration	After running through a biofiltration system located at the entrance of the greenhouse	

Water samples were either stored at −80°C until DNA extraction or processed the same day for culture- dependent techniques.

### Enumeration of Total Bacterial Populations and Specific Bacterial and Fungal Taxa in Water Samples by Culture-Dependent Techniques

Water samples were serially diluted and 0.05 mL of each dilution was placed with a spiral plater on PCA (Plate Count Agar, AES, France) for the enumeration of total bacteria and antibiotics-amended Glucose-Agar (GA) for *Bacillus* spp. (Déniel et al., 2004) while *Pseudomonas* spp. were counted on King B medium (AES, France). Enumeration was performed after a 48 h incubation period at 30°C.

For the enumeration of *Pythium* spp., *Saprolegnia* spp., and *Fusarium oxysporum* water samples were filtered through 0.45 μm cellulose acetate filters (Sartorius, Germany). The filters were placed onto 3 selective media, namely Corn Meal Agar implemented with rifampicin 0.01 g/L, ampicillin 0.25 g/L, pimaricin 0.005 g/l and pentachloronitrobenzene 0.1 g/L for *Pythium* spp., M2lev Agar implemented with two antibiotics for *Saprolegnia* spp. (penicillin 0.05 g/L and streptomycin 0.05 g/L) and Komada’s medium for *F. oxysporum* (Komada, 1975). A volume of 150 mL of water were filtered for the enumeration of *Pythium* spp. and *Saprolegnia* spp. while *F. oxysporum* was counted on two separate plates, after filtering a volume of 50 and 20 mL, respectively. *Fusarium oxysporum* propagules were also enumerated after a direct plating of 0.5 mL twice on Komada’s medium. *Pythium* and *Saprolegnia* propagules were counted after a 48 h incubation period at 25°C in the dark, whereas *F. oxysporum* propagules were counted 7 days after incubation under the same conditions. Presence of other fungal genera on such selective media was also recorded in which case identification was based on macro- and/or microscopic observations. For each taxon, enumeration was performed on 2–4 plates per water sample.

### DNA Extraction

For each sample, water was filtered through 0.2 μm cellulose acetate filters (Sartorius, Germany). Total DNA was extracted from frozen filters using FastDNA^®^ SPIN kit for soil (MP Biomedicals, Santa Ana, CA, United States) following the manufacturer’s instructions. Quality and concentration of purified DNA were determined using a UV spectrophotometer (NanoDrop1000, Thermo Fisher Scientific, United States), and dilutions of at least 10 ng/μL were prepared for each DNA sample.

### PCR Amplification and Miseq Sequencing

A total of 15 samples were selected for amplicon PCRs and Illumina Miseq PE300 sequencing, which was performed at the McGill University and Génome Québec Innovation Centre, Montréal, Canada. Amplicon libraries were constructed following two rounds of PCR amplification. The first step was performed with the PCR primers 341F (5′-CCTACGGGNGGCWGCAG-3′) and 805R (5′-GACTACHVGGGTATCTAATCC-3′) (Herlemann et al., 2011) to amplify the variable regions V3 and V4 of the *16S rRNA* gene; and primers ITS1F (5′-CTTGGTCATTTAGAGGAAGTAA-3′) and ITS4 (5′-TCCTCCGCTTATTGATATGC-3′) (White et al., 1990; Gardes and Bruns, 1993) to amplify the internal transcribed spacer. CS1 and CS2 universal adapter sequence tails (5′-ACACTGACGACATGGTTCTACA-3′ and 5′-TACGGTAGCAGAGACTTGGTCT-3′, respectively), as well as a mix of these tails with additional nucleotides (either T, AC, or TCA in 3′ edge) were added to forward and reverse primers for *16S rRNA*, and only to forward primer for ITS. All PCRs were performed with a high-fidelity polymerase including Hot Start Taq DNA polymerase from QIAGEN (Germany) for ITS amplification; and FastStart High Fidelity PCR System (from Roche but distributed by Sigma-Aldrich) for *16S rRNA* amplification, as well as for the second round of amplification (incorporation of barcode) of both amplicons.

PCR mixtures were done in 10 μL of total volume per reaction for *16S rRNA* amplification and 8 μL for ITS amplification, with the following reactive concentrations: primers 0.4 μM, MgCl_2_ 1.5 mM, DMSO 5%, dNTP 0.2 mM, Taq polymerase 0.02 U/μL, and 1 μL of environmental DNA (around 1 ng).

Amplification of *16S rRNA* was performed with an initial step at 94°C for 2 min, followed by 31 cycles of amplification at 94°C (30 s), 55°C (30 s), and 72°C (30 s), with a final extension step of 7 min at 72°C. ITS amplification was performed as follows: 96°C (15 min) followed by 35 cycles of amplification at 96°C (30 s), 52°C (30 s), and 72°C (60 s), with a final extension step of 10 min at 72°C. All amplicons were purified with the Agencourt AMPure XP system and quantified with QuantIT PicoGreen.

The second round of amplification was performed with 2 μL of purified amplicons and primers containing the Illumina adapters and indexes. PCR cycling conditions were 95°C (10 min), followed by 15 cycles of amplification (95°C for 15 s, 60°C for 30 s, 72°C for 1 min) and a final extension step at 72°C (3 min). All amplicons were purified and quantified as previously described. The purified amplicons were then pooled in equimolar concentrations, and the final concentration of the library was determined using a quantitative PCR (qPCR) NGS library quantification kit. Amplicon libraries were mixed with 10% PhiX control according to Illumina’s protocols.

### *16S rRNA* Read Filtering

The raw sequences, after Q-score filtering performed by Genome Quebec (reads with Q-score higher than 25 were kept), were processed and analyzed with QIIME v1.9.1 (Quantitative Insights Into Microbial Ecology) (Caporaso et al., 2010), according to Legrand et al. (2018) and Cobo-Díaz et al. (2019). For *16S rRNA* amplicons, the forward (R1) and reverse (R2) paired-end sequences were joined using *multiple_join_paired_ends.py*, followed by *multiple_split_libraries_fastq.py* for demultiplexing. Final length of joined sequences was approximately 460 bp. Chimera and suspicious candidates sequences (representing 12.3% of total sequences) were removed using UCHIME algorithm (Edgar et al., 2011) implemented in vsearch v1.1.3^1^ against the ChimeraSlayer database (Haas et al., 2011). Pick open strategy was used to cluster the sequences into Operational Taxonomic Units (OTUs) at 97% similarity cut-off using *pick_open_reference_otus.py* and GreenGenes v13_8 database (McDonald et al., 2012). Taxonomic assignment was performed using UCLUST algorithm (Edgar, 2010) against GreenGenes v13_8 database preclustered at 97% similarity cutoff (McDonald et al., 2012). Up to 4295 chloroplast, mitochondria and unassigned OTUs were discarded for further analysis. To minimize the inflation of rare OTUs, only OTUs with sequence count greater than 10 were included (Brown et al., 2015; Oliver et al., 2015).

### ITS Read Filtering

The raw sequences were processed and analyzed with QIIME v1.9.1 (Quantitative Insights Into Microbial Ecology) (Caporaso et al., 2010), according to Legrand et al. (2018) and Cobo-Díaz et al. (2019). The forward and reverse files were merged independently, using *multiple_split_libraries_fastq.py*. ITS1 and ITS2 regions were first extracted separately from forward and reverse fasta files respectively, using ITSx v1.0.11 (Bengtsson-Palme et al., 2013) before being concatenated in a new file. A chimera filtering, allowing to discard 7.8% of total sequences, was made on concatenated file using the UCHIME algorithm (Edgar et al., 2011) with VSEARCH v1.1.3^1^ and a modified version of the UNITE/INSDC representative/reference sequences version 7.2 (UNITE Community 2017) as reference database. The modification consisted in extracting ITS1 and ITS2 regions by ITSx software and concatenated them in the modified version of the database.

The ITS1-ITS2 concatenated file of non-chimeric sequences was used for OTU picking running the QIIME script *pick_open_reference_otus.py*, with BLAST (Altschul et al., 1990) as taxonomic assignment method and a modified version of UNITE plus INSD non-redundant ITS database version 7.1 (Kõljalg et al., 2013). The modified version consisted in concatenating ITS1 and ITS2 regions after extracting them using ITSx software. To minimize the inflation of rare OTUs, only OTUs with sequence count greater than 10 were included (Brown et al., 2015; Oliver et al., 2015).

### Alpha and Beta-Diversity Analysis

Metabarcoding datasets obtained after filtering (V3-V4 region of *16S rRNA*, ITS1-ITS2 concatenated regions) were processed equally. A single rarefaction, based on the sample with the lowest number of reads (that is, 36,900 and 15,943 for *16S rRNA* and ITS sequences, respectively), was used for alpha-diversity analysis using *single_rarefaction.py* QIIME script. Good’s coverage, OTUs richness (observed otus, Chao1 index), diversity (Shannon and Simpson indices) and evenness (dominance and equitability) were calculated with *alpha_diversity.py* QIIME script. The statistical software R version 3.5.0 was used to perform one-way ANOVA with Tukey HSD *post hoc* test, for statistical analysis. The level of significance was set at α = 0.05.

Bacterial functions were predicted by FAPROTAX (Louca et al., 2016) while fungal ecological guilds were predicted by FUNGuild v1.0 (Nguyen et al., 2016) as either pathotrophs, saprotrophs, symbiotrophs or 2 of them or all three of them. Corresponding OTU table was used for prediction of bacterial function or fungal ecological guilds as input file. Heatmaps were made using pheatmap R package with the default parameters. Relative abundance data were z-scored normalized by row. The barplots were obtained using *vegan* and RAM R-package using OTU relative abundance table (version 1.2.1.7, Chen et al., 2016). Principal Coordinates Analysis (PCoA) based on Bray-Curtis distance were performed in Calypso webtool (Zakrzewski et al., 2016) after a Hellinger-transformation of the OTU relative abundance tables and removal of OTUs with less than 0.01% of abundance. Bacterial taxa and functional abundances were first compared across water samples before entering the greenhouse (RW, GW/S, and GW/NS) using Wilcoxon-rank test and before and after biofoltration (BBF and ABF) using Deseq2 test. Fungal taxa and functional abundances were compared between RW and GW/S as well as between BBF and ABF, using Deseq2 test. Comparison of taxa abundances was performed on the 30 most abundant genera while bacterial and fungal functional groups with less than 10 sequence reads per sample were discarded from comparative analysis.

To generate PCoA plots, 2 OTU tables were used as input data including the taxonomical bacterial and fungal OTU tables generated after QIIME pipelines.

### Accession Numbers

All the raw reads have been deposited at the NCBI and are available under the Bioproject ID PRJNA531584 and PRJNA531572 for bacterial and fungal microbiota, respectively^2^.

## Results

### Microbial Enumeration

Microbial counts varied greatly across water samples (Table 2). Overall, total bacterial counts were higher in rainwater sample (RW) than in groundwater samples (GW/S and GW/NS), with approximately a 1-log bacterial count difference between the two conditions. In addition, fluorescent *Pseudomonas* spp. were not detected in the groundwater samples, while rainwater sample presented 10 UFC per mL (Table 2). Rainwater sample also included a higher level of fungal propagules including *Saprolegnia* spp. and *Cladopsorium* spp. and to a lesser extent *Mucor* spp. (Table 2).

**TABLE 2:** Enumeration of total bacterial and specific bacterial and fungal/oomycota taxa using culture-dependent techniques.

	**GW/S**	GW/NS	RW	**BBF**	**ABF**
Total bacterial population (CFU/mL)	51	20	498	1,1.10^4^	142
Fluorescent *Pseudomonas* (CFU/mL)	ND	ND	10	132	ND
*Bacillus* spp. (CFU/mL)	ND	ND	ND	320	56
*Pythium* spp. (TFU/L)	ND	ND	+	43	ND
*Saprolegnia* spp. (TFU/L)	10	27	40	3	ND
*Fusarium oxysporum* (TFU/L)	ND	ND	ND	1.10^5^	75
Additional observations		*Penicillium* spp. (+)	*Cladosporium* spp. (++)	*F. graminearum* (+)	
			*Unidentified* (++)		
			*Mucor* spp. (+)		

**FIGURE 1:**
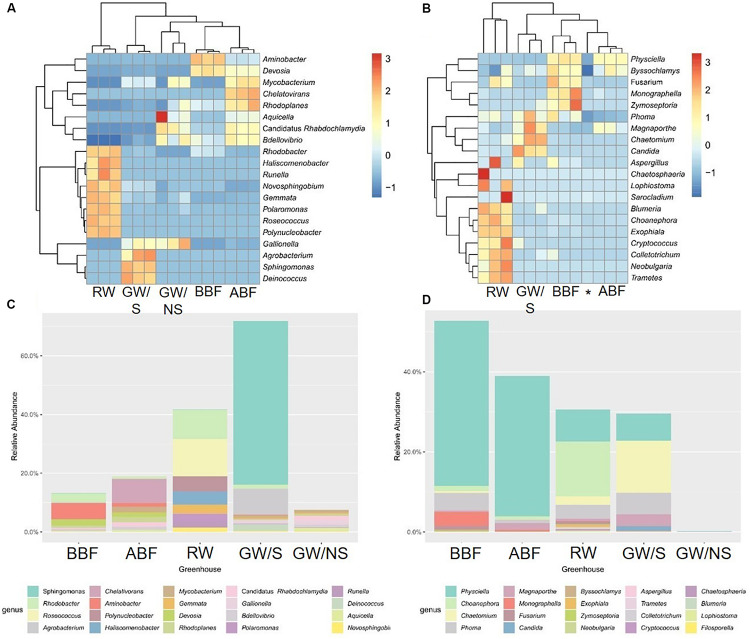
Heatmap and barplots of the 20 most abundant bacterial **(A,C)** and fungal **(B,D)** genera. The value of each of the 3 replicates (water sample code followed by A, B, or C) or the mean value is reported for heatmaps and barplots, respectively. Water sample codes: GW/S, groundwater stored in open tank; GW/NS, non-stored groundwater directly pumped out from the ground; RW, Rainwater; BBF, Before biofiltration; ABF, After biofiltration. The star refers to the GW/NS sample for fungal dataset.

**Water sample codes can be found in Figure 1 legend or Table 1; CFU, colony-forming unit; TFU, thallus-forming unit; ND, not detected; +, presence in trace amounts; ++, abundant.**

Microbial counts in water samples collected before biofiltration (BBF) were higher than after biofiltration (ABF). Biofiltration resulted in a general decrease in microbial counts, including a 2-log decrease in total bacterial counts, 1-log decrease of *Bacillus* spp. and 3-log decrease of *F. oxysporum* while fluorescent *Pseudomonas* spp., *Saprolegnia*, and *Pythium* spp. were no longer detected after biofiltration (Table 2).

### Bacterial Taxonomical and Functional Diversity Using *16S rRNA* Gene Metabarcoding

The metabarcoding of *16S rRNA* gene from 15 water DNA samples (5 water samples × 3 biological replicates) generated a total of 731,129 sequences, with between 36,900 and 58,861 sequences per sample. After filtering and rarefaction, a total of 553,500 sequences were used for further analyses, with Good’s coverage for each sample superior to 0.99. Total sequences were clustered into 3,951 OTUs assigned to 46 phyla and 125 genera.

Bacterial composition in the sample directly collected from the ground (GW/NS) was characterized by the most even distribution between OTUs while richness (represented by the number of observed OTUs or Chao1 index) was significantly lowest in both GW/NS and RW (Table 3). This latter sample was characterized by a less even distribution and more dominant genera including *Roseococcus* and *Rhodobacter* (Figure 1). The GW/S sample was moderately rich and diverse (Table 3), with the presence of dominant taxa including *Sphingomonas* and *Agrobacterium* (Figure 1). The highest bacterial richness was found in water samples from recirculating solutions (greenhouse 3), either before (BBF) or after (ABF) running through the biofiltration system (Table 3). Water samples collected after biofiltration (ABF) presented significantly higher alpha-diversity values (observed OTUs, Chao1, Shannon, Simpson and Equitability) than those collected before (BBF) while dominance was significantly lower after biofiltration (Table 3).

**TABLE 3:** Alpha-diversity indices including the number of observed OTUs, Chao1, Shannon, Simpson, dominance, and equitability indices according to various treatments for *16S rRNA* (A) and ITS dataset (B).

**A**	***16S rRNA***					
	**Observed otus**	**Chao1**	**Shannon**	**Simpson**	**Dominance**	**Equitability**
GW/S	94884^b^	113784^c^	5.0970.152^c^	0.8280.016^d^	0.1720.016^a^	0.5160.009^e^
GW/NS	507331^c^	535325^d^	7.6271.285^ab^	0.9900.009^a^	0.0100.009^d^	0.8750.026^a^
RW	5262^c^	63010^d^	5.0350.165^c^	0.9000.011^c^	0.1000.011^b^	0.5570.019^d^
BBF	135518^b^	157633^b^	6.4980.056^bc^	0.9460.002^b^	0.0540.002^c^	0.6250.006^c^
ABF	185540^a^	205937^a^	8.3410.022^a^	0.9850.001^a^	0.0150.001^d^	0.7690.004^b^

**B**	**ITS**					
	**Observed otus**	**Chao1**	**Shannon**	**Simpson**	**Dominance**	**Equitability**

GW/S	1179^c^	13214^c^	3.5780.117^d^	0.8320.027^c^	0.1670.027^b^	0.5210.023^c^
GW/NS	40^d^	75^c^	0.305^e^	0.082^d^	0.918^a^	0.057^d^
RW	3186^a^	3494^a^	5.68230.202^a^	0.9460.011^a^	0.0540.011^d^	0.6840.024^a^
BBF	31721^a^	36239^a^	5.1410.127^b^	0.8940.015^b^	0.1060.015^c^	0.6190.014^b^
A.BF	22310^b^	27225^b^	4.6800.082^c^	0.9170.005^ab^	0.0830.005^cd^	0.6000.006^b^

*Water sample codes can be found in Figure 1 legend or Table 1. Mean ± standard deviation of 3 values are reported (except for GW/NS ITS dataset for which there is one value). Different letters indicate significant differences between treatments (ANOVA, Tukey HSD test, p < 0.05).*

**FIGURE 2:**
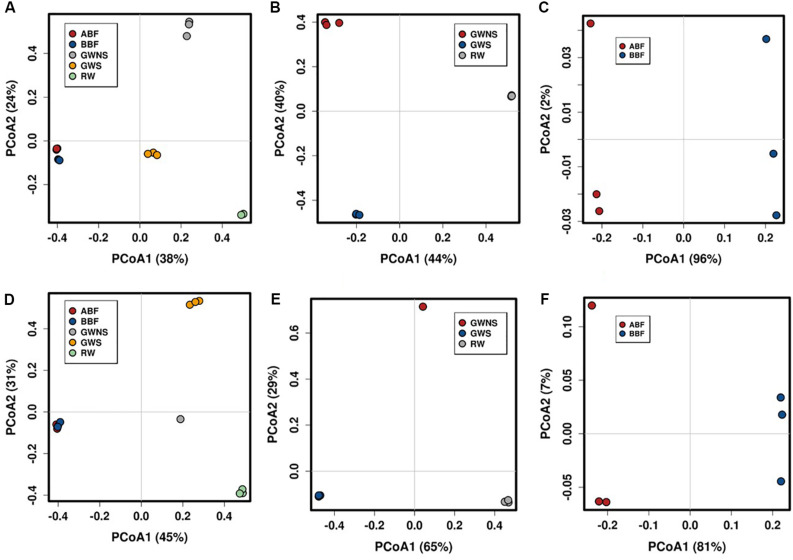
Principal Coordinates Analysis (PCoA) plots depicting differences in taxonomic composition of bacterial (above) and fungal (below) communities at OTU level. PCoA were generated by pooling all samples **(A,D)** and by separating RW, GW/S, and GW/NS into one subgroup **(B,E)** and ABF with BBF in another subgroup **(C,F)**. The relative abundance of each genus was Hellinger-transformed. The plots were obtained using Calypso webtool. Dot with the same color represents the 3 replicates of each water sample, except for GW/NS which had only one replicate for ITS dataset. Water sample codes can be found in Figure 1 legend or Table 1.

**FIGURE 3:**
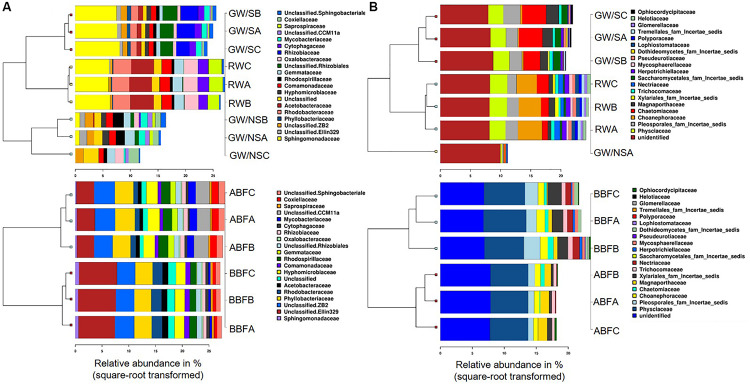
Clustered barcharts of the 20 most abundant bacterial **(A)** and fungal **(B)** families, obtained using Calypso webtool by separating RW, GW/S, and GW/NS in one subgroup (above) and ABF with BBF in another subgroup (below). The value of each of the 3 replicates (water sample code followed by A, B, or C) is reported for each water sample, except for GW/NS which had only one replicate for ITS dataset. Water sample codes can be found in Figure 1 legend or Table 1.

Principal Coordinates Analysis (PCoA) at OTU level showed that samples were differentiated from one another, except for the water samples taken before and after biofiltration (BBF and BBF) which were grouped together (Figures 2A–C). At family level, the compositional structure of GW/NS was clustered away from that of RW, which was closer to that of the microbiota of GW/S (Figure 3).

**FIGURE 4:**
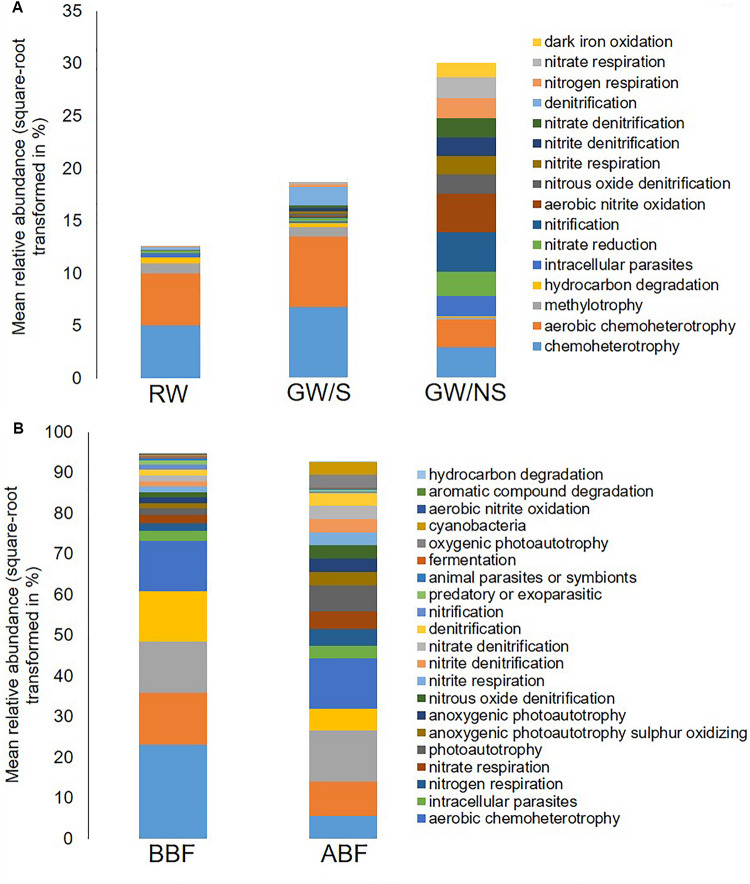
Mean relative abundance in percentage of total reads (square-root transformed) of bacterial functions assigned by FAPROTAX and significantly different from at least one other sample. Functional abundances across water samples before entering the greenhouse **(A)** and before and after biofiltration **(B)** were compared using Wilcoxon-rank test and Deseq2 test, respectively. The average value of the 3 replicates is reported for each water sample. Water sample codes can be found in Figure 1 legend or Table 1.

Significant differences in terms of taxa abundance were found. When comparing samples collected before entering the greenhouse (GW/S, GW/NS, and RW), 17 genera were found to be significantly different in at least one pairwise comparison according to Wilcoxon-rank test performed on the top 30 genera. Unclassified *Sphingomonadaceae*, *Rhodobacter*, *Roseococcus*, and unclassified *Acetobacteraceae* were the highest in the rainwater sample vs. *Sphingomonas*, *Agrobacterium*, unclassified *Phyllobacteriaceae*, *Aminobacter*, *Devosia*, and *Chelativorans* in GW/S and unclassified *koll1*, unclassified *ZB2*, unclassified *Gemmataceae*, *Gallionella*, Candidatus *Rhabdochlamydia*, *Bdellovibrio*, and unclassified *CCM11a* in GW/NS. In terms of predicted function, 16 predicted functions were significantly different in at least one pairwise comparison (Figure 4A). Sample GW/NS was enriched with predicted functions related to oxidation of dark iron and nitrogen metabolism (nitrate reduction, nitrogen-, nitrate- and nitrite respiration, nitrite-, nitrate- and nitrous-oxide denitrification, denitrification, nitrification and aerobic oxidation of nitrite) while both RW and GW/S contained highest levels of bacteria assigned to predicted functions related to chemoheterotrophy, aerobic chemoheterotrophy, methylotrophy, and hydrocarbon degradation (Figure 4A).

Among the most important differences in taxa relative abundance between samples, our results showed a significant decrease in relative abundance for 7 genera following biofiltration (*Devosia*, *Sphingomonas*, *Aminobacter, Rhodobacter*, unclassified *Ellin329*, unclassified *Phyllobacteriaceae* and unclassified *Acetabacetraceae*) and a significant increase for 12 genera (*Chelativorans*, *Gemmata*, *Rhodoplanes, Mycobacterium*, unclassified *CCM11a*, unclassified *Gemmataceae*, unclassified *Synthrophobacteraceae*, unclassified *koll1*, unclassified *Chlamydiales*, unclassified *Opitutaceae*, Candidatus *Rhabdochlamydia*, and unclassified *Rhodospirillaceae*). A significant decrease in *Agrobacterium* relative abundance was also observed with biofiltration but to a lower extent. Significant predicted functional differences were also detected after biofiltration, including a decrease in nitrification, ureolysis, aromatic compound degradation, photoheterotrophy and an increase in photoautotrophy and predicted functions related to nitrogen metabolism (including nitrate reduction, nitrogen-, nitrate- and nitrite respiration, nitrite-, nitrate- and nitrous-oxide denitrification and denitrification) (Figure 4B).

### Fungal Taxonomical and Functional Diversity Using ITS Gene Metabarcoding

The metabarcoding of the concatenated ITS1 and ITS2 regions from 13 water DNA samples (two replicates of GW/NS were discarded due to amplification problems) generated a total of 696,878 sequences, with between 15,943 and 69,640 sequences per sample. After filtration and rarefaction, a total of 207,259 sequences were used for further analyses, with Good’s coverage for each sample superior to 0.99. Total sequences were clustered into 1,092 OTUs and assigned to 6 phyla and 84 genera.

Samples collected from rainwater (RW) and before biofiltration (BBF) displayed the highest level of richness and diversity indices and GW/NS the lowest (Table 3). Unlike bacteria, fungal richness (observed OTUs and Chao1 index) and diversity (Shannon index) were significantly higher before biofiltration (Table 3).

Like bacteria, the compositional structure of fungal communities was also different among water samples, except for samples BBF and ABF, which were very close at taxonomical level (Figures 2D–F). Both water samples (BBF and ABF) were dominated by *Physciella* spp., belonging to the lichen family *Physiaceae* (Figure 1D).

## Discussion

In the present study, the fungal and bacterial diversity of water in 3 commercial hydroponic tomato greenhouses was investigated by culture-dependent and metabarcoding approaches. Our first aim was to compare microbiota in water stored in different conditions before entering the greenhouse. Secondly, we also focused on the water ecosystem in recirculating solutions, before and after running through a biofiltration system, which is commonly used in greenhouses in Brittany.

### Overall Description of the Water Microbiota

Our results revealed that the water microbiota was a very rich and diverse niche at both taxonomical and functional levels, whatever the sample. The number of bacterial OTUs varied between 507 and 1855 while that of fungal OTUs varied between 40 and 318. In line with our results, relatively high levels of bacterial richness, yielding 88 bacterial orders (vs. 141 in our case) were found in a study aiming at comparing microbiota between groundwater collected from aquifer and well ecosystems, as determined by Illumina MiSeq metabarcoding of *16S rRNA* (Korbel et al., 2017). *Burkholderiales*, *Actinomycetales*, and *Sphingomonadales* bacterial orders were found as dominant in wells and aquifers (Korbel et al., 2017); *Rhizobiales* and *Rhodobacterales* in a water drain greenhouse (López-Leal et al., 2018) and *Mycobacterium* bacterial genus and *Phoma* fungal genus in a water reuse system facility (Stamps et al., 2018). All of them were found in our study among the top 20 fungal and/or bacterial taxa. Besides, among the 11 families described as predominant in tomato growing media from soilless cultures (Grunert et al., 2016), all were found in our water microbiota before entering the greenhouse, and more specifically, the *Hypomicrobiaceae, Comamonadaceae*, and *Rhizobiaceae* families belonged to our top 20. This result suggests that such taxa encountered in growing media may partly originate from water supply.

Plant pathogen genera were found in our samples and included, among the top 20, *Agrobacterium* bacterial genus and *Fusarium*, *Monographella*, *Zymoseptoria*, *Phoma*, *Magnaporthe*, and *Colletotrichum* fungal genera, all of which include important phytopathogen species responsible for important economic loss including in greenhouses (Dean et al., 2012). Oomycetes, including *Pythium* and *Saprolegnia*, were not detected by metabarcoding simply because primer ITS1F that was used here is not a suitable forward primer for the successful amplification of oomycetes (Manter and Vivanco, 2007), with a high number of mismatches when blasted against the NCBI nt database for oomycota. In addition, OTU assigned to *Legionella* and *Bacillus cereus* (human pathogens), *Ralstonia* and *Pseudomonas* bacterial genera (both of which responsible for brown rot in plants) were also found, but to a lesser degree. The current taxa resolution granted by such metabarcoding approach allows to go down, at best, to genus level. Yet, to unequivocally identify pathogenic organisms, accurate taxonomic identification must be yielded at least to the species level given that isolates belonging to the same genus (if not species) may be either phytopathogens, non-pathogenic or could even be used as biocontrol agents. For instance, strains of *Pythium oligandrum* can be either pathogenic while other are used as antagonists to plant pathogens (Gerbore et al., 2013). In addition, presence of DNA does not imply that active communities are present in the ecosystem. Nevertheless, such metabarcoding data might be a useful tool as a first screening to narrow down the search for potential pathogenic organisms present in the ecosystem and that may be harmful. Large surveys must be undertaken to demonstrate whether metabarcoding approaches could be used as an early detection tool for pathogenic organisms and correlate such data with actual apparition of plant diseases in greenhouses. In our study, although sequences assigned to plant pathogenic genera were detected, plant diseases did not ultimately appear in commercial greenhouses.

### Impact of Water Storage Conditions

Our study also indicated that composition varied greatly across storage conditions (groundwater vs. rainwater) and among greenhouses, suggesting that water microbiota is site- and storage-condition-specific. Interestingly, water samples stored in open tanks, either coming from the rain (RW) or pumped out from the ground (GW/S), were characterized by dominant bacterial species (*Rhodobacter* and *Roseococcus* for RW and *Sphingomonas* for GW/S), unlike water directly collected from the ground (GW/NS) which displayed a more even distribution between bacterial taxa. This difference suggests that storage in open tanks may provide an environment that will ultimately lead to the adaptation of such dominant taxa. Despite a significant influence of site and storage conditions on microbiota, water samples stored in open tanks (GW/S and RW) also shared a higher degree of similarity in the structure and abundance of microbial communities, as well as in terms of bacterial function, than with the water samples directly collected from the ground (GW/NS). This indicates that a common suite of taxa and functions may underpin the microbiota associated with open-storage conditions, independently of the origin of water (rainwater or groundwater) or site.

Significant differences in terms of bacterial taxa abundances were also found between storage conditions. Differences in the composition of microbial communities implies differences in functional characteristics. Because DNA was sequenced (and not RNA or proteins), we are rather looking at the potential functions than the actual functions of the microbiota (Korbel et al., 2017), let alone that 70% of taxa could not be assigned to a bacterial function using FAPROTAX. With that in mind, we found that both water samples from open tanks were enriched with bacteria assigned to aerobic chemoheterotrophy (mainly belonging to the *Hyphomicrobiaceae*, *Flavobacteriaceae*, and *Sphingomonadaceae* families). In addition, a higher fungal diversity and/or richness was found in open-stored water than in GW/NS. Open-stored water, whatever their origin, may thus be either more subjected to contamination with aerobic chemoheterotrophs and molds (which mostly depend upon aerobic respiration for growth) and/or may provide more conducive growth conditions for such microorganisms given their direct contact with the atmosphere and their supposedly more-oxygenated environments than wells. An enrichment in methylotrophic bacteria (belonging to the *Methylobacteriaceae*, *Methylocystaceae*, and *Methylophilaceae* families) was also observed in open-stored waters. These groups of bacteria, known to colonize water, plays a key role in the biogeochemical cycle in soil ecosystem, including phosphorus acquisition, nitrogen fixation, phytohormone production, iron chelation and could be used as biofertilizers and plant growth promotion in agriculture (Kumar et al., 2016). Contrary to open-stored waters, bacterial predicted functions assigned to GW/NS were more evenly distributed, with a significant higher proportion of taxa related to nitrogen metabolism involved in nitrification or aerobic oxidation of nitrite (mainly belonging to the *Nitrospirales* order that have the potential to fix nitrogen), and in denitrification or nitrogen respiration (mainly belonging to the *Rhizobiales* and *Rhodocyclales* orders). These groups are common in terrestrial soils and are great contributors to the nitrogen cycle, involved in N removal in agricultural soils (Philippot et al., 2007; Hayatsu et al., 2008; Long et al., 2013). The higher proportion of such taxa in the water directly pumped out of the ground than in open-stored waters again suggests that either these microorganisms are entering groundwater from terrestrial source and/or that well provides an environment that is more conducive to population growth than does open-stored water (Korbel et al., 2017). Noteworthy, oxygen conditions requirements differ between denitrifying and nitrifying bacteria, with lower oxygen conditions required for denitrifying bacteria and *vice-versa*. Since both groups coexisted at high levels in the well habitat, it means that oxygen conditions may vary along time or such habitat may be sufficiently heterogeneous to generate aerobic/anaerobic interfaces, which could be hotspots for N-cycling processes including nitrification, denitrification or anaerobic oxidation of ammonium, also called anamox (Schmid et al., 2005; Nie et al., 2019). Since the latter process is not yet included in FAPROTAX database, we manually looked for anamox taxa in our OTU table and retrieved OTUs assigned to the Brocadiaceae family only in one replicate of the non-stored groundwater sample (GW/NS, 216 reads).

### Impact of Biofiltration

Bacterial diversity was significantly higher in recirculating solutions (either before or after biofiltration) than in water samples collected before entering the greenhouse, suggesting that recirculation of nutrient solutions through the irrigation system in a greenhouse may lead to enrichment of bacterial taxa. This conclusion should nevertheless be tempered by the fact that this comparison does not arise from samples collected from the same greenhouse. Both BBF and ABF samples were also dominated by *Physciella*, a genus of lichenized fungi in the family *Physciaceae*. Our results also showed that slow filtration allowed reducing cultivable bacterial loads by 2 logs while possible plant pathogens, such as *Pythium* spp. and *F. oxysporum*, used here as indicators for genus that also include plant pathogens, were also reduced. Indeed, it should be pointed out that no pathogenicity tests were performed to formally demonstrate their pathogenicity to tomato plants. Interestingly, bacterial richness and diversity was increased following biofiltration while the opposite result was found for fungi. This suggests that biofiltration may lead to enrichment of bacterial taxa and depletion in fungal taxa. While the overall fungal and bacterial compositional structure was relatively similar between both samples, metabarcoding data displayed significant differences in bacterial taxa abundance before and after biofiltration. In terms of relative abundance, these differences resulted in an increase in photoautotrophy and predicted functions related to nitrogen metabolism, except for nitrification which was depleted with biofiltration along with additional predicted functions related to photoheterotrophy, ureolysis and aerobic ammonia oxidation. However, the exact mechanisms by which some taxa would be trapped by filters while others would not remained largely unknown. Using SSCP, Renault et al. (2018) also found that bacterial population were quantitatively reduced and displayed different structures before and after biofiltration each month of sampling over the 7-month period of experiment in a tomato hydroponic greenhouse. However, taxonomic resolution only allowed to go down to phylum level. In addition, they found that differences were even more striking when slow-filters were bacteria-amended. Overall, the authors suggested that the successful removal of plant pathogenic micro-organisms relied on the microbiota diversity predominated by non-pathogens, making it difficult for phytopathogens to outcompete such complex microbiota and interaction networks.

Although preliminary, this study provides an initial insight on bacterial and fungal diversity of water introduced in commercial hydroponic greenhouse**s**. However, given the early nature of this study, additional samplings must be undertaken to take into account heterogeneity among and within sites.

Monitoring the microbiological status of water by NGS approaches may become increasingly important. Such “next-generation biomonitoring,” also termed “biomonitoring 2.0” (Leese et al., 2018) raises the possibility to detect pathogens early and identify bioindicators or taxa of interest, such as those associated with suppressive disease effects or plant growth promoting attributes, ultimately improving the assessment of ecosystem health. However, *in silico* detection of pathogens or bioindicators implies that the level of accuracy in taxa assignment must be sufficiently accurate, let alone that one species may harbor both pathogenic and non-pathogenic strains. In addition, following *in silico* identification of taxa of interest (either pathogens or beneficial), culture-dependent approaches would actually be needed to formally demonstrate such potential biological activities.

## Data Availability Statement

The datasets generated for this study can be found in the NCBI, under Bioproject numbers PRJNA531584 and PRJNA531572.

## Author Contributions

FD contributed to the conception and design of the study and performed the sampling. CD and APa performed DNA extraction and culture-dependent analysis. APi and JC-D performed read processing and OTU table filtering. APi, FL, and JC-D performed the statistical analysis. APi wrote the first draft of the manuscript. All authors contributed to the manuscript revision, read and approved the submitted version.

## Conflict of Interest

The authors declare that the research was conducted in the absence of any commercial or financial relationships that could be construed as a potential conflict of interest.

## Footnotes

1 https://github.com/torognes/vsearch

2 http://www.ncbi.nlm.nih.gov/
